# Supplementary material for: Knowledge search, knowledge integration and enterprise breakthrough innovation under the characteristics of innovation ecosystem network: The empirical evidence from enterprises in Beijing-Tianjin-Hebei region
Source: PLoS One. 2021 Dec 23;16(12):e0261558. doi: 10.1371/journal.pone.0261558 (PMC8699633; doi:10.1371/journal.pone.0261558)
Supplement: S1 File — (DOCX) [file pone.0261558.s001.docx]

**企业知识管理与创新调查问卷**

尊敬的女士/先生：

感谢您在百忙之中填写此份问卷，我们真诚地希望您能够根据自己的真实看法来回答相关问题。本调查采用无记名形式，您所填的答案仅供统计研究之用，我们对您所填写的内容绝对保密，决不个别处理或公开发表，保证不会对您造成任何不良影响。如有任何疑问和建议请与我们联系。谢谢您的帮助！

（一）企业基本情况

1．您所在企业员工人数？ 人；

2．您所在企业成立年份？ 年；

3．您所在企业的地理位置 省 市；

4．您所在企业所属行业? ；

5．您所在企业是否属于科技型企业？①是 ②否 ；

6．您所在企业属于何种性质？①国有或国有控股②民营③外资或合资④其它 ；

7．您所在企业目前的发展阶段：①初创期②成长期③成熟期 ④衰退期 ；

8．您的职位？①高层管理者 ②中层管理者 ③基层管理者 ④专业人员（研发或技术人员） ⑤其它 ；

（二）下列各项旨在了解您所在企业的**创新网络特征**情况，请您依据实际情况在适当的数字上打√，或涂颜色。

1=非常不符合；2=比较不符合；3=基本符合；4=比较符合；5=非常符合

| 网络规模 |  |  |  |  |  |
| --- | --- | --- | --- | --- | --- |
| 1.企业与政府部门产生联系的数量很多 | [1] | [2] | [3] | [4] | [5] |
| 2.企业与高校、科研机构产生联系的数量很多 | [1] | [2] | [3] | [4] | [5] |
| 3.企业与中介组织（或行业协会）产生联系的数量很多 | [1] | [2] | [3] | [4] | [5] |
| 4.企业与金融机构产生联系的数量很多 | [1] | [2] | [3] | [4] | [5] |
| 5.企业与同行企业产生联系的数量很多 | [1] | [2] | [3] | [4] | [5] |
| 6.企业与供应商产生联系的数量很多 | [1] | [2] | [3] | [4] | [5] |
| 7.企业与客户产生联系的数量很多 | [1] | [2] | [3] | [4] | [5] |
| 网络联系强度 |  |  |  |  |  |
| 8.企业与外部其他组织有长期的合作交流 | [1] | [2] | [3] | [4] | [5] |
| 9.企业与外部其他组织形成了紧密的合作关系、联系频繁 | [1] | [2] | [3] | [4] | [5] |
| 10.企业与外部其他组织的合作交往有着重要的未来规划 | [1] | [2] | [3] | [4] | [5] |

（三）下列各项旨在了解您所在企业的**创新情况**，请您依据实际情况在适当的数字上打√，或涂颜色。

1=非常不符合；2=比较不符合；3=基本符合；4=比较符合；5=非常符合

| 突破式创新 |  |  |  |  |  |
| --- | --- | --- | --- | --- | --- |
| 1.企业非常重视研发新产品或新服务 | [1] | [2] | [3] | [4] | [5] |
| 2.企业能够更快速的推出新产品或新服务 | [1] | [2] | [3] | [4] | [5] |
| 3.企业能够将突破性的技术运用于新产品或新服务的开发 | [1] | [2] | [3] | [4] | [5] |

（四）下列各项旨在了解您所在企业的**知识管理**情况，请您依据实际情况在适当的数字上打√，或涂颜色。1=非常不符合；2=比较不符合；3=基本符合；4=比较符合；5=非常符合

| 知识搜索宽度 |  |  |  |  |  |
| --- | --- | --- | --- | --- | --- |
| 1.企业能够从供应商、客户获取市场信息 | [1] | [2] | [3] | [4] | [5] |
| 2.企业能够从高校、政府部门、科研机构获取知识资源 | [1] | [2] | [3] | [4] | [5] |
| 3.企业能够从行业协会、中介组织获取知识资源 | [1] | [2] | [3] | [4] | [5] |
| 4.企业能够获取本行业的安全、技术、环境标准的信息 | [1] | [2] | [3] | [4] | [5] |
| 知识搜索深度 |  |  |  |  |  |
| 5.企业能够有效利用从供应商、客户获取的市场信息 | [1] | [2] | [3] | [4] | [5] |
| 6.企业能够有效利用从高校、政府、科研机构获取的知识资源 | [1] | [2] | [3] | [4] | [5] |
| 7.企业能够有效利用从行业协会、中介组织获取的知识资源 | [1] | [2] | [3] | [4] | [5] |
| 8.企业能够有效遵从本行业的安全、技术、环境标准 | [1] | [2] | [3] | [4] | [5] |
| 知识整合 | [1] | [2] | [3] | [4] | [5] |
| 9.企业能将获得的不同来源和不同类型的知识进行有条理分类 | [1] | [2] | [3] | [4] | [5] |
| 10.企业能够及时消化和吸收外部知识并为员工个体所掌握 | [1] | [2] | [3] | [4] | [5] |
| 11.企业能将获取的外部知识融入到企业实践并形成企业的知识体系 | [1] | [2] | [3] | [4] | [5] |
