# Supplementary material for: Knowledge search, knowledge integration and enterprise breakthrough innovation under the characteristics of innovation ecosystem network: The empirical evidence from enterprises in Beijing-Tianjin-Hebei region
Source: PLoS One. 2021 Dec 23;16(12):e0261558. doi: 10.1371/journal.pone.0261558 (PMC8699633; doi:10.1371/journal.pone.0261558)
Supplement: S2 File — (DOCX) [file pone.0261558.s002.docx]

**Questionnaire about Enterprise Knowledge Management and Innovation**

Dear lady/sir:

Thank you for filling out this questionnaire in your busy schedule. We sincerely hope you can answer relevant questions according to your true views. This questionnaire takes uninscribed forms, which means that the answers you fill in are only for statistical research. Also, we will keep the contents you fill in absolutely confidential and will not deal with them individually or publish them publicly. At the same time, we can guarantee that it will not have any adverse impact on you. If you have any questions and suggestions, please contact us. Thank you for your help!

***1. Enterprise’s Basic Information***

1. How many employees does your enterprise have? (people);
2. How long is your enterprise established? (year);
3. Your enterprise is located in (province) (city);
4. Which industry does your enterprise belong to? ;
5. Does your enterprise belong to technology enterprise?

① Yes ②No ;

1. What is the nature of your enterprise?

①State-owned or state-holding ②Private ③Foreign capital or joint venture ④Others ;

1. Which stage of development is your enterprise in?

①Initial stage ②Growth period ③Maturity period ④Recession period ⑤Others ;

1. What is your position?

①Top manager ②Middle manager ③Grassroots manager ④Professionals (R&D or technician) ⑤Others ;

***2. The following items are intended to know the characteristics of your enterprise's innovation network.***

*Please tick√ or color the appropriate numbers according to the actual situation.*

*1 represents strongly disagree; 2 represents slightly disagree; 3 represents agree; 4 represents generally agree; 5 represents strongly agree.*

| ***Network Size*** |  |  |  |  |  |
| --- | --- | --- | --- | --- | --- |
| 1. There are many connections between enterprises and government. | [1] | [2] | [3] | [4] | [5] |
| 2. There are many connections between enterprises and universities/ scientific research institutions. | [1] | [2] | [3] | [4] | [5] |
| 3. There are many connections between enterprises and intermediary organizations (or industry associations). | [1] | [2] | [3] | [4] | [5] |
| 4. There are many connections between enterprises and financial institutions. | [1] | [2] | [3] | [4] | [5] |
| 5. There are many connections between enterprises and peer enterprises. | [1] | [2] | [3] | [4] | [5] |
| 6. There are many connections between enterprises and suppliers. | [1] | [2] | [3] | [4] | [5] |
| 7. There are many connections between enterprises and customers. | [1] | [2] | [3] | [4] | [5] |
| ***Network Connection Strength*** |  |  |  |  |  |
| 8. The enterprise has long-term cooperation and connection with other external organizations. | [1] | [2] | [3] | [4] | [5] |
| 9. The enterprise has formed close cooperative relations and frequent connections with other external organizations. | [1] | [2] | [3] | [4] | [5] |
| 10. The cooperation between enterprises and other external organizations has important future plans. | [1] | [2] | [3] | [4] | [5] |

***3. The following items are designed to know the innovation of your enterprise.***

*1 represents strongly disagree; 2 represents slightly disagree; 3 represents agree; 4 represents generally agree; 5 represents strongly agree.*

| ***Breakthrough Innovation*** |  |  |  |  |  |
| --- | --- | --- | --- | --- | --- |
| 1. The enterprise attaches great importance to the development of new products or services. | [1] | [2] | [3] | [4] | [5] |
| 2. The enterprise can launch new products or services more quickly than peer enterprises. | [1] | [2] | [3] | [4] | [5] |
| 3. The enterprise can apply breakthrough technologies to the development of new products or services. | [1] | [2] | [3] | [4] | [5] |

***4. The following items are designed to know the knowledge management of your enterprise.***

*1 represents strongly disagree; 2 represents slightly disagree; 3 represents agree; 4 represents generally agree; 5 represents strongly agree.*

| ***Knowledge Search Breadth*** |  |  |  |  |  |
| --- | --- | --- | --- | --- | --- |
| 1. The enterprise can obtain market information from suppliers and customers. | [1] | [2] | [3] | [4] | [5] |
| 2. The enterprise can obtain knowledge resources from universities, government and scientific research institutions. | [1] | [2] | [3] | [4] | [5] |
| 3. The enterprise can obtain knowledge resources from industry associations and intermediary organizations. | [1] | [2] | [3] | [4] | [5] |
| 4. The enterprise can obtain the information on safety, technology and environmental standards of the industry. | [1] | [2] | [3] | [4] | [5] |
| ***Knowledge Search Depth*** |  |  |  |  |  |
| 5. The enterprise can effectively use the market information obtained from suppliers and customers. | [1] | [2] | [3] | [4] | [5] |
| 6. The enterprise can effectively use the knowledge resources obtained from universities, government and scientific research institutions. | [1] | [2] | [3] | [4] | [5] |
| 7. The enterprise can effectively use the knowledge resources obtained from industry associations and intermediary organizations. | [1] | [2] | [3] | [4] | [5] |
| 8. The enterprise can effectively comply with the safety, technology and environmental standards of the industry. | [1] | [2] | [3] | [4] | [5] |
| ***Knowledge Integration*** | [1] | [2] | [3] | [4] | [5] |
| 9. The enterprise can systematically classify different sources and types of acquired knowledge. | [1] | [2] | [3] | [4] | [5] |
| 10. The enterprise can digest and absorb external knowledge in time, which can also be mastered by individual employees. | [1] | [2] | [3] | [4] | [5] |
| 11. The enterprise can integrate the acquired external knowledge into the enterprise practice and form the enterprise’s knowledge system. | [1] | [2] | [3] | [4] | [5] |
