# Supplementary material for: Knowledge search, knowledge integration and enterprise breakthrough innovation under the characteristics of innovation ecosystem network: The empirical evidence from enterprises in Beijing-Tianjin-Hebei region
Source: PLoS One. 2021 Dec 23;16(12):e0261558. doi: 10.1371/journal.pone.0261558 (PMC8699633; doi:10.1371/journal.pone.0261558)
Supplement: S5 File — (DOCX) [file pone.0261558.s005.docx]

**The Description of Questionnaire Variables**

| **Vari-ables** | **Questions in our questionnaire** | **Specific Description** | **References** | **Reference Content/ Questions** |
| --- | --- | --- | --- | --- |
| **Netw-ork Size** | 1. There are many connections between enterprises and government.  2. There are many connections between enterprises and universities/ scientific research institutions.  3. There are many connections between enterprises and intermediary organizations (or industry associations).  4. There are many connections between enterprises and financial institutions.  5. There are many connections between enterprises and peer enterprises.  6. There are many connections between enterprises and suppliers.  7. There are many connections between enterprises and customers. | This questionnaire selects 7 indicators to measure the "network size". These indicators are based on the network size theory proposed by Hislop,. They also refer to the variable measurement method proposed by Jie X.M and Li X.D. At the same time, they combine the characteristics of technology enterprises in Beijing-Tianjin-Hebei region.   1. **Reference:** 2. These indicators draw on Hislop's definition of network scale (see the table on the right for details). 3. According to Jie X.M’ Question 2-4, Question 1-3 of this questionnaire are drawn up (corresponding one by one). 4. According to Li X.D’ Question 1-4, Question 6/2/3/7 of this questionnaire are drawn up (corresponding one by one). 5. **Innovation** 6. There are a large number of financial institutions in Beijing-Tianjin-Hebei region, which can provide strong economic support for technology enterprises. At the same time, the connection between enterprises and financial institutions will also affect innovation network size. Therefore, this questionnaire uses Question 4 to measure "network size". 7. Since a large number of Chinese technology enterprises gather in Beijing-Tianjin-Hebei region, and the connection between enterprises will also affect innovation network size, this questionnaire uses Question 5 to measure "network size". | Hislop D. The Effect of Network Size on intra-network knowledge processes. *Knowledge Management Research& Practice*, **2005**, 3, 244-252. | ***Theoretical Basis:***   1. Network size refers to the number of people involved in a network. 2. How the size of a network of actors affects the nature of intra- network social relations and knowledge processes?   As network size increases network density is likely to decrease (as it becomes problematic for the actors in such networks to retain strong ties with a significant proportion of the network’s members), which it will be suggested has significant ramifications for intra-network knowledge processes. |
|  |  |  | Jie X.M; Zuo L.L. Characteristics of collaborative innovation networks and innovation performance of firms: the mediating effect of knowledge absorptive capacity. *Nankai Business Review*, **2013**, 16, 47-56. | ***Variable Measurement:***   1. Number of enterprises in the network; 2. Number of government agencies in the network; 3. Number of scientific research institutions in the network; 4. Number of intermediary organizations (or industry associations) in the network; |
|  |  |  | Li X.D; Zhang X.Y; Hou J. The mechanism of technical standardization driving innovation performance of high-tech enterprises:the perspective of network characteristics of innovation ecosystem. *Management Review*, **2020**, 32, 96-108． | ***Variable Measurement:***   1. Number of main suppliers in the network; 2. Number of scientific research institutions in the network; 3. Number of intermediary organizations (or industry associations) in the network; 4. Number of main customers in the network; |
| **Netw-ork Con-necti-on Stren-gth** | 8. The enterprise has long-term cooperation and connection with other external organizations.  9. The enterprise has formed close cooperative relations and frequent connections with other external organizations.  10. The cooperation between enterprises and other external organizations has important future plans. | This questionnaire selects 3 indicators to measure the "network connection strength". These indicators are based on the strength of ties theory proposed by Dodgson. They also refer to the variable measurement method proposed by Jie X.M and Li X.D. At the same time, they combine the characteristics of technology enterprises in Beijing-Tianjin-Hebei region.   1. **Reference:** 2. These indicators draw on Dodgson's definition of network connection strength (see the table on the right for details). 3. According to Jie X.M’ Question 1, Question 8 of this questionnaire is drawn up. 4. We integrate Li X.D’ Question 1-3 into Question 9 of this questionnaire. 5. **Innovation**   The important future plans of enterprises and other external organizations will affect the tightness of their future cooperation and connection. Therefore, this questionnaire uses Question 10 to measure "network connection strength". | Jie X.M; Zuo L.L. Characteristics of collaborative innovation networks and innovation performance of firms: the mediating effect of knowledge absorptive capacity. *Nankai Business Review*, **2013**, 16, 47-56. | ***Variable Measurement:***   1. Long term cooperative relationship; 2. Frequent resource sharing; 3. Frequent information exchange; |
|  |  |  | Dodgson M; Gann D; Phillips N. The Oxford Handbook of Innovation Management, *Oxford: Oxford University Press,* **2013**. | ***Theoretical Basis:***   1. The measurement object of strength of ties: frequency of interaction and frequent interaction produces strong ties. 2. The relationship between strength of ties and innovation: strong ties are likely to communicate redundant information whereas weak ties are thus contexts for exploitation, with weak ties being sources of exploration. |
|  |  |  | Li X.D; Zhang X.Y; Hou J. The mechanism of technical standardization driving innovation performance of high-tech enterprises:the perspective of network characteristics of innovation ecosystem. *Management Review*, **2020**, 32, 96-108． | ***Variable Measurement:***   1. Have long-term and frequent cooperative relations with partners; 2. Mutual trust with partners; 3. Cooperation with partners is mutually beneficial; 4. A wide range of cooperation and exchange with partners (resources, information, etc.); |
| **Breakthr-ou-gh Innovatio-n** | 1. The enterprise attaches great importance to the development of new products or services.  2. The enterprise can launch new products or services more quickly than peer enterprises.  3. The enterprise can apply breakthrough technologies to the development of new products or services. | This questionnaire selects 3 indicators to measure the "Breakthrough Innovation". These indicators are based on the Exploratory Innovation theory proposed by Jansen. They also refer to the variable measurement method proposed by Fey C.F. At the same time, they combine the characteristics of technology enterprises in Beijing-Tianjin-Hebei region.   1. **Reference:** 2. These indicators draw on Jansen's definition of exploratory innovation (see the table on the right for details). 3. According to Fey C.F’ Question 6, Question 2-3 of this questionnaire are drawn up. 4. **Innovation**   Whether an enterprise attaches great importance to the development of new products or services will have an important impact on whether it can achieve breakthrough innovation. Therefore, Question 1 is used in this questionnaire to measure "breakthrough innovation". | Fey C.F; Birkinshaw J. External sources of knowledge, governance mode, and r&d performance. *Journal of Management*, **2015**, 31, 597-621. | ***Variable Measurement:***   1. Partnering with universities. How valuable are the following as sources of R&D expertise: (a) universities located close to the R&D sites, (b) universities located elsewhere in the same country, (c) universities located in other countries? 2. Partnering with alliance/JV partners. How valuable are the following as sources of R&D expertise: (a) alliance or JV partners in the same country, (b) alliance or JV partners in other countries? 3. Contracting. What percentage of the firm’s technology capability is bought or insourced from other companies? 4. Openness to new ideas. Please indicate the extent to which you agree with the following statements about your company: (a) In this company, there is a great openness to picking up ideas from outside. (b) The “not invented here” syndrome is a real problem in our company (reverse coded) . 5. Codifiability of knowledge. Which you agree with the following statements about your company: (a) New R&D personnel can easily learn their job by studying a complete set of blueprints, (b) new R&D personnel can easily learn their job by talking to experienced personnel, (c) educating and training R&D personnel is a quick and easy job, (d) a com- petitor can easily learn how to manufacture our product by studying the employees at work. 6. R&D performance. Please rate your firm’s overall per- formance during the past 3 years, in comparison with competitor firms in the industry, in terms of (a) getting new products to market quickly, (b) coming up with radical/breakthrough tech nologies, and (c) bringing breakthrough technologies to market. |
|  |  |  | Jansen J.J.P; Van D.B.F.A.J; Volberda H.W. Exploratory innovation, exploitative innovation, and performance: effects of organizational antecedents and environmental moderators. *Management Science*, **2006**, 52, 1661-1674. | ***Theoretical Basis:***  Exploratory innovations are radical innovations and are designed to meet the needs of emerging customers or markets. They offer new designs, create new markets, and develop new channels of distribution. Exploratory innovations require new knowledge or departure from existing knowledge.   1. The higher a unit’s centralization of decision making, the lower its level of exploratory innovation. 2. The higher a unit’s formalization, the lower its level of exploratory innovation. 3. There will be an inverted U-shaped relationship between a unit’s connectedness among its members and the level of exploratory innovation. 4. Environmental dynamism positively moderates the relationship between exploratory innovation and financial performance. 5. Environmental competitiveness negatively moderates the relationship between exploratory innovation and financial performance. |
| **Kno-wled-ge Sear-ch Brea-dth** | 1. The enterprise can obtain market information from suppliers and customers.  2. The enterprise can obtain knowledge resources from universities, government and scientific research institutions.  3. The enterprise can obtain knowledge resources from industry associations and intermediary organizations.  4. The enterprise can obtain the information on safety, technology and environmental standards of the industry. | According to the variable measurement method proposed by Laursen K and Su D.M, this questionnaire selects 4 indicators to measure the "Knowledge Search Breadth ". At the same time, these indicators combine the characteristics of technology enterprises in Beijing-Tianjin-Hebei region.   1. **Reference** 2. According to Laursen K’ Question 1-2, Question 1 of this questionnaire is drawn up. According to Laursen K’ Question 5-7, Question 2 is drawn up. According to Laursen K’ Question 11, Question 3 is drawn up. According to Laursen K’ Question 14-16, Question 4 is drawn up. 3. According to Su D.M’s Question 3, Question 4 of this questionnaire is drawn up. 4. **Innovation**   Intermediary organizations are also important sources for enterprises to obtain knowledge. Therefore, this questionnaire uses intermediary organizations (Question 3) to measure "Knowledge Search Breadth ". | Laursen K; Salter A. Open for innovation: the role of openness in explaining innovation performance among u. k. manufacturing firms. *Strategic Management Journal*, **2006**, 27, 131-150. | ***Variable Measurement:***  External search breadth is constructed as a combination of the 16 sources of knowledge or information for innovation listed below:   1. Suppliers of equipment, materials, components, or software 2. Clients or customers 3. Competitors 4. Consultants 5. Commercial laboratories/R&D enterprises 6. Universities or other higher education institutes 7. Government research organizations 8. Other public sector, e.g., business links, government offices 9. Private research institutes 10. Professional conferences, meetings 11. Trade associations 12. Technical/trade press, computer databases 13. Fairs, exhibitions 14. Technical standards 15. Health and safety standards and regulations 16. Environmental standards and regulations   *Notes*:   1. As a starting point, each of the 16 sources are coded as a binary variable, 0 being no use and 1 being use of the given knowledge source. Subsequently, the 16 sources are simply added up so that each firm gets a 0 when no knowledge sources are used, while the firm gets the value of 16, when all knowledge sources are used. In other words, it is assumed that firms that use higher numbers of sources are more ‘open’, with respect to search breadth, than firms that are not. 2. As a test of the robustness of the results for the measure of external search depth, we calculate an alternative measure (DEPTH COLLAB) by look- ing at whether or not the firm in question has for- mal innovation collaboration links with different external sources. This variable is based on a sub- sequent question on the U.K. innovation survey. The survey lists eight different external partners, including: (1) suppliers, (2) clients or customers, (3) competitors, (4) consultants, (5) commercial laboratories/R&D enterprises, (6) universities or other higher education institutes, (7) government research organizations, or (8) private research institutes. As in the case of BREADTH and DEPTH, the eight dummies are subsequently added up so that each firm gets a score of 0 when no partners are used, while the firm gets a value of 8 when the firm is collaborating with all potential collaboration partners. |
|  |  |  | Su D.M; Wu Z.F; Liu C. External knowledge search and its ambidexterity effect on innovation performance. *Science of Science and Management of S.& T*, **2017**, 8, 109-121. | ***Variable Measurement:***   1. The enterprise has widely used multiple search and communication channels /media for knowledge search. 2. The enterprise can search for knowledge in R&D, manufacturing, marketing and other fields. 3. The enterprise can search for knowledge in technology, management and other aspects. 4. The enterprise has acquired more knowledge in the search process. |
| **Kno-wled-ge Sear-ch Dept-h** | 5. The enterprise can effectively use the market information obtained from suppliers and customers.  6. The enterprise can effectively use the knowledge resources obtained from universities, government and scientific research institutions.  7. The enterprise can effectively use the knowledge resources obtained from industry associations and intermediary organizations.  8. The enterprise can effectively comply with the safety, technology and environmental standards of the industry. | According to the variable measurement method proposed by Laursen K and Su D.M, this questionnaire selects 4 indicators to measure the "Knowledge Search Depth". At the same time, these indicators combine the characteristics of technology enterprises in Beijing-Tianjin-Hebei region.   1. **Reference** 2. According to Laursen K’ Question 1-2, Question 5 of this questionnaire is drawn up. According to Laursen K’ Question 5-7, Question 6 is drawn up. According to Laursen K’ Question 11, Question 7 is drawn up. According to Laursen K’ Question 14-16, Question 8 is drawn up. 3. According to Su D.M’s Question 3, Question 8 of this questionnaire is drawn up. 4. **Innovation**   Whether an enterprise can effectively obtain knowledge from intermediary organizations is an important indicator to measure the "search depth" of enterprises. Therefore, this questionnaire uses intermediary organizations (Question 7) to measure "Knowledge Search Depth". | Laursen K; Salter A. Open for innovation: the role of openness in explaining innovation performance among u. k. manufacturing firms. *Strategic Management Journal*, **2006**, 27, 131-150. | ***Variable Measurement:***  External search depth is defined as the extent to which firms draw intensively from different search channels or sources of innovative ideas. It is constructed using the same 16 sources of knowledge as those used in constructing External search breadth.   1. Suppliers of equipment, materials, components, or software 2. Clients or customers 3. Competitors 4. Consultants 5. Commercial laboratories/R&D enterprises 6. Universities or other higher education institutes 7. Government research organizations 8. Other public sector, e.g., business links, government offices 9. Private research institutes 10. Professional conferences, meetings 11. Trade associations 12. Technical/trade press, computer databases 13. Fairs, exhibitions 14. Technical standards 15. Health and safety standards and regulations 16. Environmental standards and regulations   *Notes*:  Each of the 16 sources are coded with 1 when the firm in question reports that it uses the source to a high degree and 0 in the case of no, low, or medium use of the given source. As in the case of breadth, the 16 sources are subsequently added up so that each firm gets a score of 0 when no knowledge sources are used to a high degree, while the firm gets the value of 16 when all knowledge sources are used to a high degree. Again, it is assumed that firms that use higher numbers of sources are more ‘open’ with respect to search depth than firms that are not. |
|  |  |  | Su D.M; Wu Z.F; Liu C. External knowledge search and its ambidexterity effect on innovation performance. *Science of Science and Management of S.& T*, **2017**, 8, 109-121. | ***Variable Measurement:***   1. The enterprise strongly and intensively uses some specific search channels for knowledge search. 2. The enterprise can deeply search and extract knowledge in specific fields such as R&D, manufacturing and marketing. 3. The enterprise can deeply search and extract knowledge in specific aspects such as technology or management. 4. The enterprise can deeply search and use the knowledge in specific fields such as R&D or manufacturing or marketing. 5. The enterprise can deeply search and extract knowledge in specific aspects such as technology or management. |
| **Kno-wled-ge Integratio-n** | 9. The enterprise can systematically classify different sources and types of acquired knowledge.  10. The enterprise can digest and absorb external knowledge in time, which can also be mastered by individual employees.  11. The enterprise can integrate the acquired external knowledge into the enterprise practice and form the enterprise’s knowledge system. | According to the variable measurement method proposed by Cummings J.N and Zhan X.D this questionnaire selects 3 indicators to measure the "Knowledge Integration". At the same time, these indicators combine the characteristics of technology enterprises in Beijing-Tianjin-Hebei region.  **Reference**   1. According to Cummings J.N’ Question 1, Question 10 of this questionnaire is drawn up. According to Cummings J.N’s knowledge classification, Question 9 is drawn up. 2. According to Zhan X.D’s Question 3, Question 9 of this questionnaire is drawn up. According to Zhan X.D’s Question 1, Question 10 is drawn up. According to Zhan X.D’s Question 4, Question 11 is drawn up. | Cummings J.N. Work groups, structural diversity, and knowledge sharing in a global organization. *Management Science*, **2004**, 50, 352-364. | ***Variable Measurement:***  *Question:*   1. On average, how often did you share each type of knowledge during the project with group members (1—never; 2—rarely; 3—sometimes; 4—regularly; 5—a lot)? 2. On average, how often did you share each type of knowledge during the project with nongroup employees inside your division, nongroup employees outside your division, or the customer(1—never; 2—rarely; 3—sometimes; 4—regularly; 5—a lot)?   Notes: type of knowledge: (a) General overviews (e.g. project goals, milestone estimates, or member responsibilities); (b) Specific requirements (e.g. numerical projections, market forecasts, or order requests); (c) Analytical techniques (e.g. statistical tools, detailed methods, or testing procedures); (d) Progress reports (e.g. status updates, resource problems, or personnel evaluations); (e) Project results (e.g. preliminary findings, unexpected outcomes, or clear recommendations). |
|  |  |  | Zhan X.D. Structural capital and technological capability:the effect of external knowledge management and knowledge distance. *Science & Technology Progress and Policy*, **2019**, 36, 144-152. | ***Variable Measurement:***   1. The enterprise can digest and absorb external knowledge, transform the knowledge of other organizations into the knowledge mastered by individual employees, as well as transform individual knowledge into enterprise knowledge base. 2. There are no obstacles for other cooperative organizations to transfer their technology and knowledge to our enterprises. 3. The enterprise can systematically classify different sources and types of knowledge, and then gradually master it. 4. The enterprise can integrate the acquired external knowledge into the practical process of solving problems and form its own knowledge system. |
