# Supplementary material for: Knowledge search, knowledge integration and enterprise breakthrough innovation under the characteristics of innovation ecosystem network: The empirical evidence from enterprises in Beijing-Tianjin-Hebei region
Source: PLoS One. 2021 Dec 23;16(12):e0261558. doi: 10.1371/journal.pone.0261558 (PMC8699633; doi:10.1371/journal.pone.0261558)
Supplement: Attachment — Submitted filename: Response to Reviewers 2.0.docx [file pone.0261558.s006.docx]

**Response to Reviewers**

**Dear Editor:**

**PLOS ONE:**

Re: Edits requested on your submission PONE-D-21-27668R1 - [EMID:19f0d2c30a1aad94]

Please find attached a revised version of our manuscript originally entitled “Knowledge search, knowledge integration and enterprise breakthrough innovation under the characteristics of innovation ecosystem network——the empirical evidence from enterprises in Beijing-Tianjin-Hebei region”.

Thanks very much for your careful review, which helps us improve the quality of our manuscript.

**Editor’s suggestion**: “Please ensure that you refer to Table 9 in your text as, if accepted, production will need this reference to link the reader to the Table.”

**Response:** we determine to use Table 9 in our manuscript, and write the reference to link the reader to the Table. After checking the full text in detail, we found that according to the questions raised by reviewer 1 in the last review comments, that is “As for the variables used, it is indicated that they were obtained from the responses to the questionnaire survey. However, there is no specific mention of what kind of questions were actually used”, we supplemented “Table 2: Variable Measurement and Source” in our manuscript. However, we are very sorry that we did not update other forms in time. **After modification, our text content and each table can be matched one by one** (see the manuscript for details)**.**

Please review again. Thank you very much!

Yan-Li Zhang, Dan-Tong Wang, Long Xu

Nov 23^rd^, 2021
